# Epicardial TGFβ and BMP Signaling in Cardiac Regeneration: What Lesson Can We Learn from the Developing Heart?

**DOI:** 10.3390/biom10030404

**Published:** 2020-03-05

**Authors:** Esther Dronkers, Manon M. M. Wauters, Marie José Goumans, Anke M. Smits

**Affiliations:** Department of Cell and Chemical Biology, Leiden University Medical Center, 2300 RC Leiden, The Netherlands; e.dronkers@lumc.nl (E.D.); manonwauters96@gmail.com (M.M.M.W.); m.j.goumans@lumc.nl (M.J.G.)

**Keywords:** Epicardium, TGFβ, BMP, EMT, invasion, cardiac development, cardiac repair

## Abstract

The epicardium, the outer layer of the heart, has been of interest in cardiac research due to its vital role in the developing and diseased heart. During development, epicardial cells are active and supply cells and paracrine cues to the myocardium. In the injured adult heart, the epicardium is re-activated and recapitulates embryonic behavior that is essential for a proper repair response. Two indispensable processes for epicardial contribution to heart tissue formation are epithelial to mesenchymal transition (EMT), and tissue invasion. One of the key groups of cytokines regulating both EMT and invasion is the transforming growth factor β (TGFβ) family, including TGFβ and Bone Morphogenetic Protein (BMP). Abundant research has been performed to understand the role of TGFβ family signaling in the developing epicardium. However, less is known about signaling in the adult epicardium. This review provides an overview of the current knowledge on the role of TGFβ in epicardial behavior both in the development and in the repair of the heart. We aim to describe the presence of involved ligands and receptors to establish if and when signaling can occur. Finally, we discuss potential targets to improve the epicardial contribution to cardiac repair as a starting point for future investigation.

## 1. Introduction: The Epicardial Timeline

The epicardium, a mesothelial layer covering the heart, has been recognized as a distinct structure for a long time, whilst its origin and function remained uncertain. Until halfway through the 20th century, the general consensus was that epicardial cells originated from the myocardium. Even though Kurkiewicz [1] discovered in 1909 that epicardial cells derive from an extra-cardiac source, we know today as the pro-epicardium (PE), this finding was neglected for several decades until it was confirmed by Manasek in 1969 [2]. In the 30 years following, the epicardium has been described by multiple observational studies in various animal species ranging from quail to hamster [3,4,5]. Nevertheless, it took until the end of the 20th century for the functional role of the epicardium to be revealed; retrovirally-labeled PE cells were traced over time and were eventually observed within the myocardium, suggesting that the epicardium contributes cells to the developing heart [6,7]. A comparable labeling approach showed that epicardial cells within the epicardium undergo epithelial to mesenchymal transition (EMT), migrate into the heart and locally become vascular smooth muscle cells and cardiac fibroblasts [8]. Over the last years, extensive research established that epicardial cells are for instance involved in stabilization of the vasculature [9], formation of the conduction system [10], and the process of myocardial compaction [11,12]. The importance of the epicardium was demonstrated when formation of the epicardium was impeded, causing severe cardiac effects such as a thin-walled myocardium and defective coronary vasculature [13,14]. 

In the adult mammalian heart, the epicardium as a single mesothelial cell layer displays little activation. Interestingly, this ‘quiescence’ is not necessarily indefinite. An important observation was made in 2006 when Lepilina et al. found that injury reactivated the epicardial layer in the zebrafish heart. This process resembled embryonic epicardial activation, and was shown to be essential for regeneration of the adult zebrafish heart [15]. To further assess the potential of the adult epicardium, in vitro models were established for adult mouse [16], and human [17] epicardial cells. These cell culture models revealed that adult cells retain the capacity to undergo EMT. However, in vivo these activation processes of EMT and subsequent migration and cell type differentiation seem to occur less efficient in the adult heart compared to the developing heart. For instance, in the adult heart there appears to be limited migration of epicardial derived cells into the injured myocardium [18,19]. This observation created a window of opportunity to boost the epicardial response to improve cardiac regeneration, which has emerged as an area of active research.

During cardiac repair in organ-regenerating animals, e.g., the newt and zebrafish, several vital processes such as cardiomyocyte proliferation [20], and blood vessel formation [21] are governed by recapitulation of developmental processes. This is in line with the general tissue regeneration paradigm that initiation of a fetal gene program is at the foundation of the endogenous regenerative response, as was also shown for e.g., mammalian axons and bone [22,23]. Therefore, to fully understand how the epicardium can be exploited to improve repair of the injured heart, it is important to comprehend its behavior during development and to unravel the signals steering this process. A signaling pathway that is involved in many processes underlying epicardial behavior is the transforming growth factor (TGF) β pathway. This family of growth factors, well known for its role in EMT and invasion of cells of different origins [24], is considered to be important for epicardial cell behavior during development and is therefore a potential target for rejuvenating the adult epicardium. The aim of this review is to provide one of the first comprehensive overviews of current knowledge of TGFβ family signaling in the embryonic epicardium and to discuss to what extend this response is recapitulated in the injured adult heart. We aim to achieve this in a structured approach, discussing the presence of ligands, associated receptors, and signaling for distinct components of the signaling family. Finally, we will discuss potential targets to improve the epicardial contribution to cardiac repair as a starting point for future investigation.

## 2. TGFβ Family Signaling Overview

**Figure 1:**
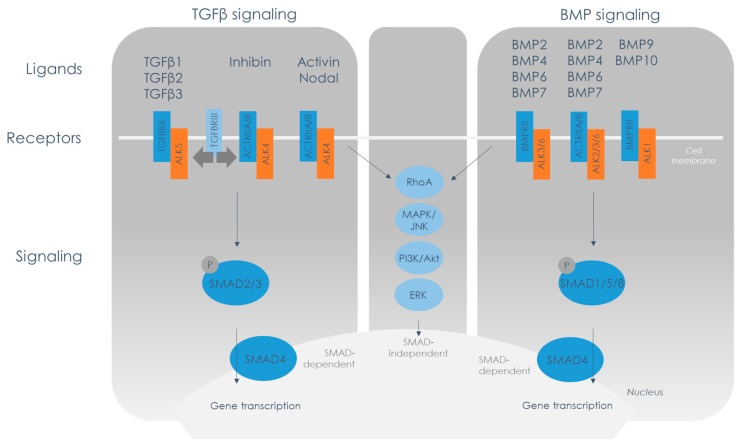
Schematic overview of TGFβ family signaling. TGFβ family signaling can be divided into two clusters: TGFβ- (left) and BMP- (right) signaling. TGFβ1, -2 and -3 bind the TGFβ receptor II and activate signaling via type I receptor ALK5. Activin, nodal and inhibin can bind to the activin receptor IIA or IIB and propagate signaling via ALK4. Signaling via ALK4 and ALK5 leads to phosphorylation of SMAD2/3. Upon binding to SMAD4, this SMAD complex translocates to the nucleus to initiate gene transcription. Of note is that ALK7 (not displayed) can also initiate signaling via SMAD2/3, but this type I receptor is assumed to be unimportant in epicardial behavior. On the BMP side, BMPs can bind to either the activin receptor IIA or IIB, or to the BMP type II receptor, that can activate the signaling cascade via ALK2, -3 or -6, or via ALK1. Signaling via ALK1/2/3/6 results in phosphorylation of SMAD1/5/8 which, after binding to SMAD4, translocates to the nucleus and starts gene transcription. Besides the described SMAD-dependent pathways, both TGFβ and BMP pathways can activate SMAD-independent pathways, shown in the middle panel.

The TGFβ family is a large group of proteins named after the cytokine that was described first [25], the polypeptide presently known as TGFβ1. The family has multiple members including TGFβs, bone morphogenetic proteins (BMPs), activins/inhibins, and growth and differentiation factors (GDFs). The TGFβ family components are critical during development and disease as its proteins are involved in the regulation of essential cellular processes including proliferation, differentiation, adhesion, and apoptosis (reviewed in [26]). Signaling occurs via an intricate system of ligands, receptors, and intracellular molecules. The TGFβ family can roughly be divided into two clusters: a TGFβ cluster and a BMP cluster, which are schematically depicted in Figure 1. TGFβ and activin ligands bind to specific receptor combinations that induce the phosphorylation of SMAD2/3, while BMPs bind to receptor combinations that result in phosphorylation of SMAD1/5/8. The phosphorylated SMADs form a complex with SMAD4 and translocate to the nucleus to induce gene transcription (see Figure 1). Multiple mechanisms ensure that the signaling cascade is highly controlled at all levels, which we will discuss separately below.

TGFβ family ligands are secreted as a complex where the active growth factor is bound to a prodomain. TGFβ family members can signal in a autocrine or paracrine fashion. Upon secretion, TGFβ ligands can be stored in the extracellular matrix (ECM) by binding to heparin, or to latent TGFβ binding protein (LTBP), thereby preventing the association of the ligand with its receptor and controlling ligand bioavailability (reviewed in [27]). Activation of the latent TGFβ ligands, more specifically TGFβ1–3, may be controlled by release of the pro-domain by the extracellular environment [28], for example by furin-mediated cleavage [29]. Additionally, as shown for BMP7, the pro-domain can also be replaced by the type II receptor enabling the receptor to bind to the growth factor domain [30]. However, the presence of the BMP pro-domain does not compromise the biological activity of several of the BMP ligands, including BMP4, 5, 7, 9 and BMP10 [31,32,33].

After the ligands are released from the ligand binding proteins, they bind to a TGFβ family receptor complex (reviewed in [34]). These receptors are subdivided into three groups: type II receptors (TGFβRII, BMPRII, ACTR2A or ACTR2B), type I receptors (activin receptor like kinase (ALK) 1-7) and type III receptors (endoglin and TGFβR3). Each ligand of the TGFβ family binds to a specific combination of type I and a type II receptors, which are both essential for downstream signaling. More specifically, the ligand binds two type II receptors and two type I receptors, in order of highest affinity, to ultimately form a heterotetrameric complex. Subsequently, the type II receptor kinase activates the type I receptor. The type I receptor propagates the signal from the membrane to the nucleus by phosphorylation of receptor-mediated SMADs (R-SMADs). There are two main signal transduction cascades: either via phosphorylation of SMAD2 or -3 or via phosphorylation of SMAD1, -5 or -8. Hence, the type I receptors are subdivided into two clusters: the TGFβ cluster, in which the type I receptors ALK4 or ALK5 propagate the signal via phosphorylation of SMAD2/3, and the BMP cluster, whose type I receptors ALK2, -3 or -6 phosphorylate SMAD1/5/8 (see Figure 1). The phosphorylation of R-SMADs initiates the formation of a complex that consists of two R-SMADs and one common SMAD (co-SMAD), which is also called SMAD4 (reviewed in [35]). After translocation of the R-SMAD/SMAD4 complex into the nucleus, SMADs interact with DNA and other transcription factors to regulate gene expression. Besides receptor regulated SMADs, there are inhibitory SMADs (I-SMADs), SMAD6 and SMAD7, that negatively regulate TGFβ signaling through competing with the R-SMADs for type I receptor binding and via regulation of receptor degradation (reviewed in [36]), allowing for yet another level in signal regulation.

Of course, there are exceptions to the general division in a TGFβ and a BMP cluster; in some cases, BMPs can induce SMAD2/3 phosphorylation or TGFβ can induce SMAD1/5/8 phosphorylation [37,38]. This can be due to an aberrant composition of type I receptors in the heterotetrameric complex, e.g., TGFβ ligand can initiate SMAD1/5/8 phosphorylation when the type I receptor complex consists of a heterodimer of ALK5 and ALK1 [39]. This cross-phosphorylation can be due to the presence of TGFβ type III receptors in the receptor complex. These accessory receptors have a transmembrane domain, similar to the type II receptors, but lack the intracellular kinase domain that is essential for activation of the type I receptor. Type III receptors are therefore not able to initiate signaling themselves, but can modulate signaling by type II receptors.

Besides signaling in a SMAD-dependent manner, TGFβ is also able to exert biological effects by activation of SMAD-independent pathways (see Figure 1). One of these pathways is Rho GTPase signaling, which is known for its role in actin reorganization, an essential part of EMT. Other pathways involved are MAPK associated, including ERK, JNK, and p38 MAPK, and the PI3K/Akt pathway (reviewed in [40]). Activation of SMAD-independent pathways can be provoked by both TGFβ and BMP ligands. 

Overall, the TGFβ family of signaling components provides a broad range of responses that can be regulated on multiple levels. The highly regulated availability of ligands, and the presence of specific combinations of receptors on the cell surface determine if a cell is able to activate the signaling cascade. On top of this, type III receptors and interaction with SMAD-independent pathways can modulate the signal.

## 3. Epicardium in Development and Disease

**Figure 2:**
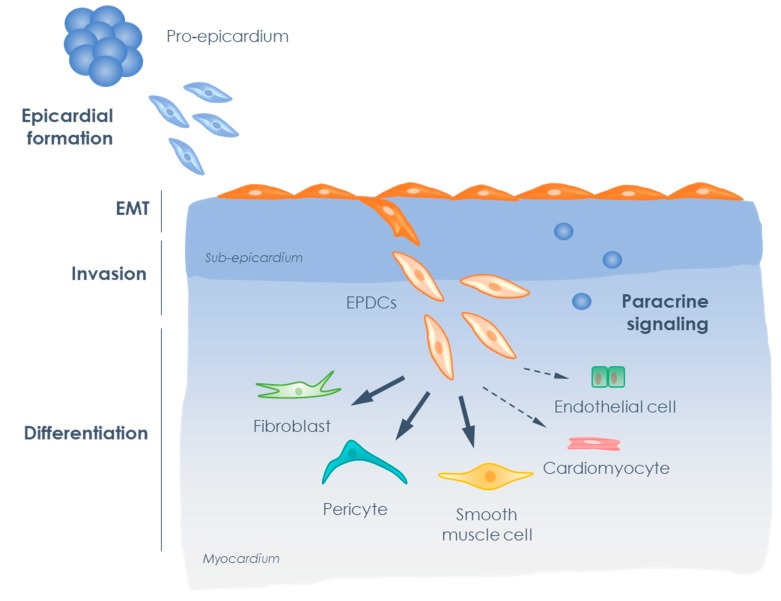
Schematic overview of epicardial behavior during development. Pro-epicardial cells migrate towards the heart and cover it to form the epicardium. When the epicardium has enveloped the heart, cells start to undergo epithelial to mesenchymal transition (EMT). This allows the epicardial derived cells to invade into the heart and differentiate into various cell types, mainly cardiac fibroblasts, smooth muscle cells, and pericytes. In addition, epicardial cells secrete paracrine factors that contribute to the development of the heart.

As mentioned previously, the epicardium plays an essential role in the formation of the heart. Cardiac development starts with the formation of a bilayered linear heart tube consisting of a myocardial layer lined by the endocardium on the luminal side. The heart tube follows a sequence of looping events during embryonic day 9 (E9) in the mouse, which corresponds to week 3–4 of human development, ultimately resulting in the formation of a four chambered heart. From E9.5 onwards, cells from the pro-epicardium (PE), a cell cluster located at the venous inflow tract, migrate towards the heart and proliferate to cover the bare myocardium with a third cardiac layer: the epicardium [41]. By E11.5, the epicardial cells have enveloped the heart and at this point display a cuboidal epithelial phenotype [42], characterized by the expression of cell adhesion molecules such as E-cadherin and β-catenin, that ascertain integrity of the layer and the maintenance of an apical-basal polarity [43]. Furthermore, the epicardial cells express Wilms’ tumor 1 (WT1) and retinaldehyde dehydrogenase 2 (RALDH2) [44], proteins that are often used as markers for activated epicardium. Then, a subset of the activated epicardial cells undergoes EMT [5,45] (Figure 2). During EMT, the cells lose their epithelial phenotype by disruption of their cell adhesion profile and rearrangement of their cytoskeletal organization, enabling them to detach from neighboring cells and become motile. Additionally, the cells acquire functional mesenchymal characteristics, such as the ability to secrete extracellular matrix (ECM) proteins. The main result of epicardial EMT is that it enables the cells to invade the sub-epicardial layer and migrate into the myocardium [46] (Figure 2). After migration, the epicardial derived cells (EPDCs) can differentiate into different cell types (reviewed in [47]). They predominantly become interstitial fibroblasts and adventitial fibroblasts, which are important for proper organization of the myocardial wall [48], and smooth muscle cells (SMCs), which will cover the vessels and are crucial for vascular maturation [49]. Differentiation of EPDCs into endothelial cells and cardiomyocytes during cardiac development has been reported, but is likely to occur very rarely (reviewed in [47]). A special role for EPDCs has been postulated in the formation of the atrioventricular (AV) junction, the annulus fibrosus [50], and the AV valves [51] (reviewed in [52]). Besides the cellular contribution of EPDCs to the developing heart, EPDCs also have a paracrine contribution by secreting factors that are essential for the developing myocardium. For instance, epicardial derived retinoic acid [11,53] and fibroblast growth factors (FGFs) [54] are important for cardiomyocyte proliferation, while epicardial GATA4/GATA6 signaling regulates endothelial cell recruitment that is indispensable for coronary plexus development [55].

In the adult heart, the epicardium does not display epicardial activation markers WT1 and RALDH1 and 2 [18,56], suggesting that it is a quiescent layer. However, the epicardium can be awakened by ischemic injury, resulting in thickening of the epicardial layer and sub-epicardial mesenchyme [56,57]. The post-injury epicardial activation is accompanied by recapitulation of developmental characteristics, such as the upregulation of epicardial activation genes and EMT markers [15,18,56]. Whether adult EPDCs, like their fetal counterparts, migrate into the injured myocardium, is still under debate (reviewed in [47]) but appears to be less prominent in the adult heart [18]. Importantly, the significance of epicardial re-activation upon injury was underlined by studies demonstrating that preventing epicardial expansion and EMT resulted in a worse cardiac outcome after ischemia-reperfusion in mice [58]. Conversely, increasing epicardial EMT and migration by thymosin β4 treatment resulted in improved cardiac outcome in the injured mouse heart [57].

Appreciating that the epicardium can be stimulated to improve cardiac regeneration led to a focus on pathways involved in the main features of epicardial behavior: EMT and invasion. As was introduced before, key players in regulating these processes are members of the TGFβ family. Given the distinction between TGFβ and BMP signaling, we describe their possible regulation within the epicardium separately, starting with TGFβ signaling.

## 4. TGFβ Signaling in Epicardial Behavior during Cardiac Development

### 4.1. Expression of TGFβ Members in the Epicardium during Development

**Figure 3:**
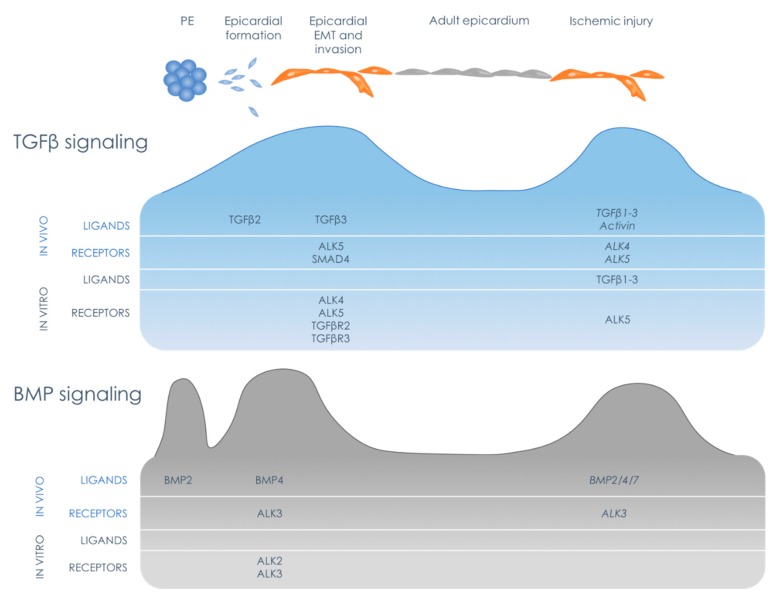
Schematic overview of TGFβ and Bone Morphogenetic Protein (BMP) signaling activity during the different stages of epicardial behavior. At the top, a timeline of epicardial activity is indicated, starting with the pro-epicardium (PE) and pro-epicardial migration towards the heart, followed by formation of the epicardium, epicardial EMT and invasion, subsequently epicardial quiescence in the healthy adult heart and ultimately the epicardial reactivation in the injured adult heart. For every stage, the known expression levels of ligands and receptors in vivo and in vitro are specified, based on the literature described in the main text. Expression levels determined in zebrafish are noted in italic. Based on the expression levels, a prediction of the activity of respectively TGFβ and BMP signaling over time is displayed by the curvature.

The first requirement for TGFβ signaling to occur in the epicardium is the presence of the actual proteins. There are three TGFβ ligands, TGFβ1, -2, and -3, that are quite similar in structure and function. However, each of them has a specific spatiotemporal gene expression pattern during cardiogenesis [59] (see Figure 3). In the mouse, the first ligand to be expressed in relation to the epicardium is TGFβ2. *Tgfb2* mRNA is present as early as E9.5 in the PE and remains detectable in the first epicardial cells that appear on the outside of the myocardium at E10.5. A clear epicardial mRNA expression pattern of *Tgfb2* is maintained until E12.5, after which it starts to decline. A similar expression pattern was observed in the epicardium of chick embryos at a comparable developmental stage [60]. *Tgfb3* is not observed anywhere in the heart at early developmental stages. However, from E11.5 onwards, when the epicardium is established and starts to participate in the formation of the heart, *Tgfb3* mRNA expression increases and is pan-epicardially expressed [59]. TGFβ3 has also been observed in the epicardium of 3 week old rat pups, suggesting a persistent epicardial expression in the neonatal epicardium [61]. In contrast to the epicardial expression of *Tgfb2* and -*3*, no obvious expression for *Tgfb1* was reported in the ventricular epicardium, but *Tgfb1* mRNA was found to be localized to the epicardium of the AV sulcus [59]. Remarkably, it was found that all three TGFβ ligands are highly expressed in the epicardium lining the AV sulcus and outflow tract, suggesting they play a role in this region. In summary, TGFβ2 is expressed during early heart development when the epicardium is formed (E9.5–E12.5); while TGFβ3 is more likely to be involved in later phases, when the epicardium contributes to cardiogenesis (E11.5–onwards).

Since TGFβ can signal in both an autocrine and paracrine fashion, the expression observed in the epicardial region does not necessarily result in actual signaling within the epicardium. To that end, the presence of the associated receptors is required to be able to determine if a cell is susceptible for signaling. Unfortunately, literature regarding receptor expression in the epicardium is scarce, which might be related to the limited availability of specific antibodies, the very low expression levels, or simply the fact that the epicardium is often overlooked in cardiac research. Interestingly, in vitro studies did reveal that mouse epicardial cells in culture do not express the type I receptor *Alk7* but have high levels of *Alk4* and *Alk5* [62]. Furthermore, cultured chick epicardial cells express *TGFBR2* and *ALK5*, suggesting that TGFβ signaling in epicardial cells can occur [60]. The fact that mouse embryos with an epicardial specific knock-out of *Alk5* [63] or *Smad4* [64] display an aberrant phenotype indicates that *Alk5* and *Smad4* are present in the developing mouse heart.

TGFβ ligands are present, suggesting an important role in epicardial behavior. However, since these ligands can be stored in a latent form in the extracellular matrix of the heart, protein expression does not automatically correlate with spatiotemporal pathway activation. Therefore, determining where phosphorylated SMAD2/3 or other downstream targets are localized within the epicardium would provide better insight into which cells are involved in actual signaling.

### 4.2. Functional Role of TGFβ in the Epicardium during Development

To determine if the expression of TGFβ members is functionally relevant for cardiac development, multiple conventional knock-out (KO) animals have been generated which are almost all embryonically lethal. Interestingly, severe cardiac defects were only observed in *Tgfb2* deficient embryos. These embryos exhibited a spectrum of cardiac malformations including ventricular septum defects, early trabeculae formation, dual outlet of the right ventricle and dual inlet of the left ventricle [65,66]. All other TGFβ related KO embryos did not display lethal cardiac defects. *Tgfb3* deficient mice died after birth due to cleft palate [67] and abnormal lung development [68]. Interesting to note is that in the review of Azhar et al., they describe that *Tgfb3* mutant mice displayed signs of segmentally thick epicardium and an underdeveloped myocardium, indicating that before birth an epicardial defect is present [69]. *Tgfb1* deficient embryos displayed inflammatory disease and impaired hematopoiesis, and died of compromised vasculogenesis. Any observed cardiac malformations in these embryos are likely an indirect effect of defective vasculogenesis [70,71]. *Alk5*-/− embryos [72] and *Tgfbr2*−/− embryos [73] displayed phenotypes comparable to the *Tgfb1* deficient embryos, suggesting that these proteins are all involved in the same developmental processes. Since TGFβ is critical in a wide variety of developmental mechanisms, it is not surprising that KO mouse models exhibit a range of defects of which it is difficult to determine cause and consequence. However, three conclusions can be drawn. Firstly, there is a specific role for *Tgfb2* in cardiogenesis. Secondly, the distinct phenotypes of the KO models of the three ligands demonstrate that TGFβ ligands are not interchangeable in vivo, probably due to the differences in spatiotemporal expression patterns and affinity for receptor binding. And thirdly, the defects observed in *Tgfb2*−/− and *Tgfb3*−/− mice are related to developmental processes where epithelial-mesenchymal interactions occur, e.g., cochlear morphogenesis in the inner ear [65], respiratory epithelial cell differentiation, and palatal fusion [68], while malformations in *Tgfb1*−/− are more related to impaired growth and inflammation [74,75,76].

Since TGFβ is crucial for multiple cellular processes in the embryo, cell specific KO studies are essential to determine the role of TGFβ signaling specifically in the epicardium. A more specific model in this regard is a transgenic mouse line expressing *Cre*-recombinase under control of an epicardial-restricted fragment of the *Gata5* promoter [53,77]. This *Gata5-Cre* mouse was crossed with a floxed *Alk5* mouse line to specifically knock-out *Alk5* in epicardial cells as early as in the PE at E9.5 [53]. The embryos displayed a properly formed epicardium indicating that ALK5 signaling is likely dispensable for epicardium formation [63], an observation that was also done in chick pro-epicardial explants [78]. In contrast to normal formation of the epicardium, epicardial related defects were observed in these epicardial-specific *Alk5* deficient mouse embryos at E12; they displayed a detached epicardial layer, a diminished smooth muscle cell coverage of the coronary vessels, and a thinner myocardial wall due to reduced cardiomyocyte proliferation [63]. This phenotype is in agreement with disturbed epicardial function, which during normal development will contribute to vessel coverage and thickening of the myocardium [11]. Hence, this observation suggests that ALK5, although not vital for epicardial formation, is essential in epicardial behaviour in vivo. When looking at the propagation of TGFβ and BMP signaling at the level of the SMADs, an inducible KO of *Smad4* specifically in cells expressing the epicardial marker WT1 did not induce severe cardiac malformations in the mouse embryo. Interestingly, these embryos displayed a reduced number of EPDCs and cardiac fibroblasts [64], indicating compromised epicardial EMT and invasion. The fact that the overall cardiac phenotype was not severely affected in this KO embryo, suggests that SMAD-independent signaling pathways are also involved in epicardial behavior.

The in vivo studies suggest that both ALK5 and SMAD4 are essential for epicardial behavior, yet through an unresolved mechanism. To further unravel the involvement of TGFβ signaling in the different aspects of epicardial behavior, in vitro models are important to be able to independently investigate epicardial EMT and epicardial invasion. Various in vitro models are used to study epicardial EMT. For instance, chick epicardial cells were studied by transferring hearts to a collagen gel allowing the epicardial cells to grow out as a monolayer [60]. After removal of the heart, stimulation with TGFβ ligand initiated EMT in the epicardial monolayer, demonstrated by a morphological change and an increase in EMT markers [60]. The same approach was used to show that TGFβ induces EMT in mouse epicardial cells derived from E12.5 embryonic hearts [79]. Epicardial cells were also derived from human fetal hearts [80]. Instead of epicardial outgrowth of cardiac tissue specimens, these human fetal cells were isolated by digestion of the isolated epicardial layer, followed by culture of the resulting single cell suspension in an epicardium specific culture medium. Stimulating these human fetal epicardial cells with TGFβ resulted in the induction of EMT [81]. Interestingly, the KO of *Alk5* [63] or the use of an ALK4/5/7 kinase inhibitor [60,79,81] prevents EMT of epicardial cells in chick, mouse, and human cell culture models. This finding translates to the in vivo observation that at the moment that epicardial cells should start to undergo EMT (E12), the epicardium-specific *Alk5* KO embryo presents with an aberrant epicardial phenotype. The observed detached epicardial layer could be due to the absence of a sub-epicardial mesenchyme as a result of deficient EMT. Whether the *Alk5* total KO embryo displays a detached epicardial layer and absent coronary vessel coverage remains elusive, as it has not been described. In the *Tgfb1*−/− and *Tgfb2*−/− embryos, the vascular phenotype and overall symptoms are likely to be too severe to determine an epicardial specific defect. Interestingly, in the *Tgfb3*−/− embryo an abnormal epicardial layer and immature myocardium were reported [69], which is in line with the observations in vitro.

Following EMT, epicardial cells acquire the ability to invade underlying tissue. Invasion of epicardial cells can be studied in more detail in ex vivo cultured hearts. For instance, when ex vivo E12.5 mouse embryonic hearts with YFP-labeled WT1+ cells were stimulated with TGFβ, the number of YFP+ cells invading the myocardium increased [82]. In a similar approach, this time using cultured avian hearts, TGFβ was shown to induce epicardial invasion in a dose-dependent manner [83]. Moreover, in vitro studies suggest that TGFβ signaling via ALK5 is essential in this regard. For instance, mouse embryonic epicardial cells display an increased invasion in a Boyden chamber assay when stimulated with TGFβ or transduced with constitutively active (ca)ALK5 [79]. Furthermore, viral induction of caALK5 in chick heart explant cultures also induced epicardial invasion into a collagen gel [60]. Overall, this data confirms the in vivo observation that TGFβ and its signaling is crucial for epicardial behavior, and specifically that ALK5 signaling is involved in the induction of both epicardial EMT and invasion. 

There are studies reporting that TGFβ does not induce epicardial EMT and invasion, and even inhibits FGF-induced epicardial EMT and invasion in a chick heart model [84]. They describe a contraction of epicardial cells and the formation of a cell mass in response to TGFβ. However, this process of contraction was in a later publication related to loss of epithelial markers [60]. Furthermore, mesenchymal differentiation and invasion of epicardial cells might be dependent on variations in cell culture conditions. In this case, the deviant observations could be due to the absence of fetal bovine serum (FBS) and the relatively high concentration of TGFβ (10 ng/mL), which is different from other studies about epicardial EMT. Furthermore, culture conditions such as cell layer confluency can influence the EMT response [80]. Interesting to note is that in contrast to in vivo studies, where different TGFβ ligands have distinct and uninterchangeable functions, in vitro there does not seem to be a difference between the types of TGFβ; all three TGFβ ligands elicited a similar effect on EMT and invasion in the majority of the studies [60,79,80]. 

To conclude, although there are some opposing findings in literature, likely due to technical differences, there is abundant evidence that in the epicardium TGFβ induced ALK5 signaling is essential for cardiac development by regulating epicardial EMT and invasion of epicardial cells. 

### 4.3. Downstream Signaling Mechanisms of TGFβ in the Epicardium during Development

When the TGFβ pathway is activated, several cell biological mechanisms are set in motion. These involve orchestrating the process of EMT- including changes in cell adhesion molecules and the cytoskeleton-, and regulating invasion of transformed mesenchymal cells into the underlying tissue. 

The regulation of cell adhesion molecules in epithelial cells is a well-known effect of TGFβ stimulation [85]. In the context of the epicardium, Dokic et al. demonstrated that within 30 min after addition of TGFβ, the membrane expression of the junctional proteins E-cadherin and β-catenin started to decline, suggesting reduced cell-cell adhesion [83]. The effect of TGFβ on intercellular strength was further demonstrated by mechanically disrupting an epicardial monolayer and determining the number of resulting fragments as a measure of the weakness or strength of cell-cell adhesions. The fragment count was much higher in TGFβ-treated epicardial monolayers, demonstrating diminished intercellular strength. The epicardial monolayers were also treated with the soluble form of the α4β1 integrin ligand Vascular Cell Adhesion Molecule 1 (sVCAM1), which prevented TGFβ-induced changes in E-cadherin and β-catenin [83] and therefore maintained the epithelial cell adhesion profile. Indeed, incubation with soluble VCAM-1 in combination with TGFβ reduced the number of fragments compared to TGFβ alone [83]. Interestingly, incubating chick hearts with sVCAM-1 prevented TGFβ induced epicardial EMT in vitro and epicardial invasion ex vivo [83]. This experiment underlines that changes in cell adhesion molecules are essential for epicardial EMT to occur. Given the fact that TGFβ regulates the presence of cell adhesion molecules, it is likely that an important step in TGFβ initiated EMT is downregulation of cell adhesion molecules.

Concomitantly to alterations in the cell adhesion molecule profile, the cytoskeleton undergoes dramatic changes during the process of EMT. The regulation of the cytoskeleton is often related to TGFβ-induced Rho signaling [60,79,83,86,87], known for its role in actin dynamics and cytoskeletal behavior of the cell (reviewed in [88]). Indeed, Rho activity increased rapidly when rat epicardial derived cells were stimulated with TGFβ [83]. Furthermore, exposure of the cells to a p160 Rho kinase inhibitor (Y27632) prevented the TGFβ-induced formation of smooth muscle markers in chick [60] and mouse [79,86] epicardial cells. Furthermore, an activator of Rho signaling, lysophosphaditic acid (LPA), achieved a similar degree of epicardial cell invasion into chick hearts as TGFβ ligand stimulation [83]. However, one may need to take into account that LPA can also activate TGFβ signaling [89], so additional research is necessary to confirm that Rho activation induces epicardial invasion. Together, these data imply the involvement of the Rho pathway, in TGFβ signaling initiated epicardial EMT.

Changing the cell-adhesion profile and cytoskeleton ultimately results in the transition of an epithelial to a mesenchymal cell type, after which cells are primed to become motile and invade the underlying tissue. Motility can be regulated by many factors, for example *Mylk*, *Rock1* and *Tmsb4x*. The combination of these factors has been described as an epicardial motile gene program [90]. Interestingly, it was found that TGFβ stimulation resulted in the nuclear accumulation of myocardin related transcription factors (MRTFs), essential factors in the motile gene program. In fact, viral overexpression of MRTF-A led to exaggerated epicardial invasion in an ex vivo cultured embryonic mouse heart [90], overall suggesting that TGFβ signaling can induce MRTFs which in turn are sufficient to induce epicardial invasion. Furthermore, TGFβ was shown to activate invasion via the SMAD-independent MAPK-ERK pathway. In this process, the TGFβ-dependent production of hyaluronic acid (HA) by hyaluronan synthase 2 (Has2) was essential. The produced HA binds the CD44 receptor and induces upregulation of vimentin and the initiation of invasion [91].

Overall, TGFβ signaling appears to orchestrate a range of factors that ultimately result in enhanced motility of the epicardial cell.

### 4.4. The Role of Activin Signaling in Epicardial Behavior during Cardiac Development

So far, we mainly described epicardial TGFβ signaling through the ALK5 receptor, a process that leads to phosphorylation of SMAD2/3. Although ALK5 is predominantly described in this context, it is not the only receptor resulting in SMAD2/3 phosphorylation. As depicted in Figure 1, SMAD2/3 can also be phosphorylated via ALK4. This type I receptor dimer forms a complex with the activin receptor IIa (ACVR2A) or IIb (ACVR2B) that can initiate signaling upon binding with activins, inhibins or nodal.

Not much is known about the role of activin signaling in cardiac development or regarding the epicardial expression of activin. Most of the KO mouse models related to activin signaling do not have a clear cardiac phenotype (reviewed in [92]). An exception to this is the type II receptor ACVR2B KO mouse, which displays severe morphological cardiac malformations such as septal defects, abnormal patterning of the outflow tract, and aberrant positioning of the great arteries [93]. Furthermore, nodal has been linked to impaired left-right patterning of the heart and several anatomical defects, such as the tetralogy of Fallot [94]. Embryos devoid of the ALK4 receptor are lethal in an early developmental stage due to disrupted primitive streak formation, and it is therefore not possible to assess its role in cardiac development in this genetic model. Some suggestion may however come from in vitro data. Firstly, *Alk4* was shown to be expressed in epicardial cells in vitro [62], implying that signaling via this receptor is a possibility. Secondly, multiple studies have shown that the ALK4/5/7 kinase inhibitor SB431542 can prevent epicardial EMT in vitro [60,79,81] pointing towards ALK4, 5 or 7 as main type I receptors, of which ALK5 is the most common. The importance of specifically ALK5 for epicardial EMT and invasion has been shown in vitro and in vivo (as described in Section 4.2) [63]. However, a potential role for ALK4 may not be neglectable, as ALK4 signaling has been implied in EMT of several cell lineages, mainly in the field of oncology [95,96,97,98,99], but also in endocardial EMT [100]. More research will be required to determine if signaling via ALK4 plays a role in epicardial behavior.

## 5. BMP Signaling in Epicardial Behavior Cardiac Development

As schematically shown in Figure 1, the other side of the TGFβ family consists of the BMP pathway that can be activated by BMPs and signals via SMAD1/5/8. While initially known for its ability to induce bone formation [101], BMP signaling emerged as a central regulator of several important processes during development and disease. In the context of cardiac development, the BMP pathway is involved in the specification of the epicardial lineage in the pro-epicardium. The ratio between BMP2 and FGF2 expression was shown to determine if the pro-epicardial cell differentiates towards the myocardial lineage, in case of high levels of BMP2, or the epicardial lineage, in case of high levels of FGF2 [102]. When directed towards the epicardial cell lineage, pro-epicardial cells migrate towards the heart. To be able to do this, pro-epicardial cells are thought to undergo EMT in order to migrate towards the heart and form the epicardium. Interestingly, pro-epicardial EMT is regulated via the type I receptor ALK2, suggesting a potentially different mechanism in pro-epicardial EMT compared to epicardial EMT which is mainly regulated via ALK5 [78].

In the developing heart, BMP ligands are mainly expressed in the myocardium [103]. They are especially highly expressed in the AV myocardium, indicating a role in the development of the AV canal [103]. Indeed, KO studies for BMP related proteins (reviewed in [104]) revealed that both the *Bmp2* deficient mouse and cardiomyocyte specific *Alk3* KO mouse displayed defects in the formation of the AV canal and also in the formation of the AV valves [103,105,106]. This was at least in part caused by impaired endocardial EMT [103,107]. Mouse embryos with a specific KO of *Alk3* in *Wt1*+ epicardial cells displayed abnormalities in the tissues of the AV junction as well [108], suggesting that in addition to the endocardium, epicardial BMP/ALK3 signaling may also be involved in the formation of the AV region. In contrast, in these embryos with epicardial specific knock-down of ALK3, neither the development of the pro-epicardium, the formation of the epicardium, nor the migration of EPDCs into the myocardium were affected [108]. Recently, BMP signaling was described to be involved in epicardial maturation [42]. Epicardial maturation is the process that starts after the epicardium has formed, during which the cells turn from a cuboidal into a squamous epithelial cell layer, characterized by flattening of the cells and elongation of the nuclei [42]. In WT1KO embryos, this process is impaired, resulting in persistence of cuboidal cells, which coincided with a higher expression of epicardial BMP4 compared to wildtype embryos. Addition of BMP inhibitor LDN-193189 rescued the phenotype of WT1KO embryos, revealing that BMP signaling needs to be absent for proper epicardial maturation [42] (see Figure 3).

The epicardial specific *Alk3*−/− embryos did not show any defects in epicardial EMT in the myocardium but revealed a potential role for BMP/ALK3 signaling in epicardial EMT in the AV region. The effect of BMP signaling on EMT was further corroborated by in vitro experiments. In murine epicardial cells, expression of both type I receptors ALK2 and ALK3 was abundant, while ALK6 levels were hardly detectable [62]. Treatment with BMP2 resulted in the loss of epithelial character in murine epicardial cells. Interestingly, contrary to TGFβ, BMP2 did not promote smooth muscle cell differentiation. An inhibitor of ALK2 and ALK3 could prevent BMP2 induced EMT. To further investigate which type I receptor is involved in EMT, epicardial cells were treated with caALK2, caALK3 or caALK5. Cells treated with caALK2 and caALK5 showed an increase in smooth muscle cell markers, while EMT induced by caALK3 did not result in SMC differentiation [62]. This finding suggests that BMP2-induced EMT signals via ALK3 and SMAD1/5/8 phosphorylation, and leads to a distinct differentiation pathway than TGFβ induced EMT via SMAD2/3 phosphorylation (see Figure 1). Given the phenotype of the epicardial specific *Alk3*−/− embryo, one could speculate that BMP/ALK3 predominantly regulates epicardial EMT in the AV junction and annulus fibrosus, giving rise to epicardial derived fibroblasts that are essential for the formation of the slow-conducting properties of this region.

Overall, developmental epicardial BMP signaling was shown to participate in pro-epicardial EMT, the maturation of the epicardium, and the formation of the AV sulcus. Interestingly, often the absence of BMP signaling appears to be essential for proper regulation of these processes, indicating that epicardial BMP signaling is dependent on very strict spatiotemporal expression of BMP ligands and receptors (see Figure 3).

## 6. The Role of Accessory Receptors Endoglin and TGFβR3 in Developmental Epicardial Behavior

Besides for the type II and type I receptors of the TGFβ family, an emerging role has been described for the two TGFβ type III receptors endoglin and TGFβR3. These structurally related proteoglycans reside in the cell membrane, but cannot initiate the TGFβ signaling pathway themselves due to the lack of a kinase domain. However, they can participate in signaling by presenting ligands to type II receptors.

The accessory receptor endoglin is mainly known for its contribution to angiogenesis [109,110]. In the developing heart, endoglin is expressed in endothelial cells and in mesenchymal cells of the AV canal [111,112]. A potential role for endoglin in EMT was suggested in a study where knock-down of endoglin by siRNA in atrioventricular (AV) canal explants of the chick embryo reduced ALK5-mediated EMT during cardiac valve formation [111]. Furthermore, endoglin was shown to be essential for EMT in kidney cancer cells as reduction of endoglin levels by shRNA increased epithelial markers and reduced mesenchymal markers [113]. Similar to other mesenchymal cells, human epicardial cells increase the expression of endoglin after EMT. A direct role for endoglin in this process was shown by blocking endoglin with an antibody in epicardial cells, which increased epithelial markers E-cadherin and VCAM-1 [114]. However, it could not prevent TGFβ induced EMT, indicating that this process is not depending on endoglin. These data show that endoglin, besides its well-known function in angiogenesis, can be a driver of EMT in the heart and might be an interesting topic to investigate further in an epicardial context.

The other accessory receptor, TGFβR3 or β-glycan, modulates TGFβ signaling by binding a ligand and presenting it to the type II receptors, thereby increasing the TGFβ responsiveness of the cell [115]. Although TGFβR3 is able to bind to TGFβ1, TGFβ3, BMPs [116] and inhibin [117], the presence of TGFβR3 is especially necessary to allow TGFβ2 binding to the TGFβRII, as this normally occurs with very low affinity [115,118,119]. Besides ligand presentation, TGFβR3 can also modulate TGFβ signaling via its cytoplasmic domain [120].

Within the heart, TGFβR3 is expressed in both the myocardium and epicardium [121], which is already suggestive for a role for TGFβR3 in cardiac development. Indeed, the TGFβR3 KO mice are embryonically lethal at E14.5 due to impaired coronary vessel formation. Interestingly, these embryos also display an aberrant epicardial phenotype, demonstrated by an excessively thick sub-epicardial layer containing blood islands, coinciding with a thin myocardial compact layer [121], while vascular smooth muscle cell recruitment to the coronary vessels appeared to be normal. This phenotype is different from the epicardium-specific *Alk5*−/− embryo, which displayed defective SMC coverage [63], indicating that TGFβR3 has a non-redundant role next to ligand presentation to the TGFβRII. Another explanation could be that in the absence of TGFβR3, TGFβ1 and -3 can still initiate signaling via ALK5, as these ligands are less dependent on the presence of TGFβR3 compared to TGFβ2. TGFβ1 and -3 could therefore partially compensate for the loss of TGFβ2 signaling, which is not possible in the *Alk5* KO.

In contrast to endoglin, the function of TGFβR3 in the epicardium has been investigated extensively using epicardial cells isolated from murine embryonic hearts (E11.5) deficient of TGFβR3 or their wildtype littermates [79,122]. Cultured *Tgfbr3*-deficient murine epicardial cells expressed reduced levels of mesenchymal markers SM22α and vimentin [123], but were able to lose their epithelial phenotype when stimulated with TGFβ1, TGFβ2 [122] or BMP2 [62]. Furthermore, these cells were able to acquire smooth muscle cell (SMC) characteristics when stimulated with TGFβ1 or TGFβ2 [122]. Therefore, TGFβR3 does not appear to be vital for epicardial EMT in vitro. However, epicardial cells deficient for *Tgfbr3* do display a decreased migration capacity [123] and diminished invasion potential upon stimulation with either TGFβ1, TGFβ2 or BMP2 in a modified Boyden chamber assay [62,122]. For epicardial invasion, the cytoplasmic domain of TGFβR3 was found to be essential [122]. This domain is not involved in ligand presentation to the TGFβRII [120], suggesting that TGFβR3 regulated epicardial invasion in a different manner. Several mechanisms have been proposed to be involved in TGFβR3 guided epicardial invasion. Firstly, the cytoplasmic domain of TGFβR3 has a 3 C-terminal amino acids domain, STA, that can bind GIPC (GAIP-interacting protein, C terminus). While *Tgfbr3*-deficient cells treated with a construct for full length *Tgfbr3* demonstrated rescued invasion, *Tgfbr3* lacking the 3 C-terminal acids could not restore invasion in KO cells [122]. Moreover, an siRNA against GIPC abolished TGFβ/BMP2 induced invasion in wildtype cells, demonstrating that GIPC is essential [62,122]. However, overexpression of GIPC1 in *Tgfbr3*−/− cells could not rescue the *Tgfbr3*−/− phenotype, indicating that the complex formation with GIPC is indispensable for TGFβR3 to induce epicardial invasion [122]. Secondly, TGFβR3 was shown to be involved in TGFβ-initiated activation of the RhoA pathway, which is essential to induce epicardial invasion. Knock-down of RhoA resulted in ligand-independent epicardial invasion, demonstrating that RhoA needs to be absent for epicardial invasion to occur. The impaired invasion in TGFβR3−/− cells could partially be restored by overexpression of a dominant negative RhoA, suggesting that TGFβR3 is involved in the reduction of RhoA that is required for epicardial invasion. This was shown to signal via Par6 and Smurf1 [87]. Lastly, a RNA sequencing experiment comparing ligand-stimulated *Tgfbr3*+/+ and *Tgfbr3*−/− epicardial cells found that NFκB signaling is differently regulated in *Tgfbr3*−/− cells compared to controls in vitro, independent of the ligand used [124]. Additional research confirmed that NFκB activity is reduced in ligand-stimulated *Tgfbr3*−/− cells and that ligand-induced invasion of *Tgfbr3*+/+ cells can be prevented when NFκB is inhibited [124,125]. Binding of β-Arrestin 2 (β-Arr2) to the cytoplasmic domain of TGFβR3 is suggested to be involved in this effect, for its described function in NFκB inhibition in breast cancer cells [126]. Indeed, preventing TGFβR3 to bind to β-Arr2 promoted epicardial invasion in the absence of a ligand [125].

The exact modulation of epicardial TGFβR3 in TGFβ and BMP signaling seems to be complex, as it is bound by an array of ligands, but can also be reduced by several inhibitors. For instance, both inhibitors of ALK4/5/7 and ALK2/3 were able to block TGFβR3 mediated invasion in response to either TGFβ2 or BMP2 respectively [62]. Moreover, TGFβR3 is also essential for FGF2 [122] and high molecular weight hyaluronic acid [122] induced epicardial invasion. Therefore, the best fitting hypothesis seems to be that TGFβR3 is an essential element in a broad spectrum of factors regulating epicardial invasion. For instance, the TGFβR3 could bring the TGFβ and FGF receptors in spatial vicinity by binding its ligands, and subsequently initiate crosstalk between FGF and TGFβ signaling that is known to be involved in processes like EMT [127].

## 7. TGFβ Family in the Epicardial Response to Cardiac Injury

### 7.1. Epicardial TGFβ Signaling in the Injured Heart

Thus far, we established a potential contribution of TGFβ and BMP signaling in the epicardium during heart development. However, as described previously, the epicardium is also involved in the injured heart. Cardiac injury arises when an occlusion of the coronary vasculature results in cardiac ischemia and damage of downstream tissue. The contribution of TGFβ signaling in the repair of the injured adult heart has been described extensively. Upon infarction, the protein expression of TGFβ ligands in the mouse heart increases. TGFβ1 and TGFβ2 expression is fast and decreases after 3–7 days, while TGFβ3 expression is induced after a few days and remains for a longer period [128]. Concomitantly, levels of pSMAD2 increase within 24 h in the injured mouse heart [129,130], while one of the inhibitory SMADs, SMAD7, decreases [129], pointing towards increased TGFβ signaling. As the widespread availability of TGFβ ligands indicates, TGFβ is involved in multiple processes in the injured heart, such as immunoregulation, ECM regulation and fibrosis (reviewed in [131,132]). It has been shown that the epicardium is also involved in the repair program of the murine and zebrafish heart [15,58]. Hence, knowing that TGFβ and BMP signaling are of importance in the embryonic epicardium raises the question if the TGFβ family is also involved in the epicardial behavior of the injured heart.

To be able to study adult epicardial EMT and invasion, in vitro cell culture models using patient derived epicardial cells were developed. In these human adult cells, it was established that all three TGFβ ligands are expressed, as well as the type I receptor ALK5 [114], indicating that cells are able to respond to TGFβ ligands. When these cells were stimulated with TGFβ, protein levels of the EMT marker Snail increased, as well as the mesenchymal marker Smooth Muscle Actin. Furthermore, cells treated with TGFβ underwent a phenotypical switch from cobblestone epithelial-like cells towards spindle-shaped mesenchymal cells. This shows that adult epicardial cells in culture retain the ability to undergo EMT in response to TGFβ treatment [114], similar to the embryonic situation. In addition, TGFβ-induced EMT could be prevented by the ALK4/5/7 kinase inhibitor SB431542, indicating that EMT is initiated via ALK4 or ALK5 signaling. Interestingly, incubation of adult epicardial cells with soluble VCAM-1 prevented TGFβ-induced EMT in adult epicardial cells [114], an observation that was also done in the embryonic epicardium [83] (see Section 4.3), demonstrating that fetal mechanisms are recapitulated in the re-activated adult epicardium. On the other hand, a role for Rho signaling could not be established in adult epicardial EMT [114], which could suggest that this pathway, although involved in fetal epicardial cells, is shut down in adult epicardial cells. Additional research is needed to confirm this observation. Interestingly, in a direct comparison between fetal and adult human epicardial cells, it was shown that adult cells are intrinsically less prone to undergo EMT than human fetal EPDCs [81]. This indicates that although the epicardium of the adult heart is still able to undergo EMT, this process might be less efficient in comparison to the fetal heart. So far, the underlying cause of this intrinsic difference is not known, but one could speculate that enhanced levels of endogenous VCAM-1 or E-cadherin, or reduced levels of TGFβ receptors can decrease the susceptibility of epicardial cells to undergo EMT. 

Thus far, in vitro studies indicate that TGFβ can regulate epicardial EMT via ALK5. Limited knowledge is available on epicardial TGFβ signaling and repair in vivo. It has been shown that the activated adult epicardial cells undergo EMT and form a sub-epicardial layer [56]. From developmental studies, we know that the TGFβ family is involved in the regulation of these processes (see Section 4), suggesting that TGFβ could also be involved in epicardial behavior in the adult heart. Moreover, combining data from various studies suggest that TGFβ signaling could be relevant in the epicardium of the injured heart. For instance, one of the few studies describing expression levels of TGFβ members specifically in the epicardium showed that Raldh2+ epicardial cells within the infarcted area of the zebrafish heart were positive for *tgfβ1*, *-2* and *-3* 14 days post-cryoinjury (dpci). Furthermore, strong mRNA expression of *alk5b* was present in the injured heart 4 dpci, and appeared to be specifically enhanced in the epicardium. This expression decreased over time, but was maintained up to 14 dpci. Although not described by the authors, mRNA expression of *alk4* appeared to be present in the epicardium as well at 4 dpci. The presence of relevant ligands and receptors indicates that epicardial TGFβ signaling can take place in the injured heart. Blocking TGFβ signaling with the ALK4/5/7 inhibitor SB431542 resulted in defective regeneration of the damaged tissue. This cannot be directly attributed to the epicardium because besides epicardial expression, injury induces an overall increase of pSmad3 in the zebrafish heart indicating a widespread activation of TGFβ signaling [133]. The impaired regeneration was attributed to impaired proliferation of cardiomyocytes [133]. In addition, the formation of ECM was heavily disrupted. No effect of the TGFβ inhibitor was observed on the expression pattern of Raldh+ epicardial cells [133], indicating that there is no direct effect on epicardial behavior. However, one could argue that the observed defects in regeneration could be partly introduced by the absence of TGFβ signaling in the epicardium. Firstly, because cardiomyocyte proliferation in the developing heart is dependent on epicardial secretion of factors. Secondly, because the epicardium is a contributor to the formation of the ECM in the regenerating heart, e.g., by delivering essential ECM proteins such as fibronectin [134] and ColXII [135]. Interestingly, the expression pattern of ColXII in the injured zebrafish heart changed upon treatment with the TGFβ inhibitor; while this protein was present in both the epicardium and the wounded area of the control injured heart, the ColXII protein was absent in the wounded area upon treatment with a TGFβ inhibitor, and was only present in epicardium [135]. Since epicardial derived cells secrete matrix proteins [134,135], the absence of ECM producing cells in the wound area could point to defective epicardial behavior, for instance caused by impaired EMT and migration. 

Since SB431542 inhibits ALK4/5/7 signaling, its effect on the repair of the zebrafish heart can also be attributed to defective activin/ALK4 signaling. This is supported by the finding that a zebrafish KO of *inhbaa*, a subunit of Activin, results in less CM proliferation in the regenerating zebrafish heart [136], similar to treatment with SB431542. This was regulated via Alk4 and Smad3. A role for the epicardium was not implied in this study, but expression of *inhbaa* in the injured heart appeared to be mainly in the epicardial and endocardial layer [136], which could suggest that the phenotype of the *inhbaa* KO is regulated via, among others, the epicardium.

Therefore, although so far no direct link between the epicardium and the diminished regeneration of the zebrafish heart after inhibition of TGFβ signaling could be established, combination of the expression levels of TGFβ members in the epicardium (see Figure 3) with the functional effects observed in zebrafish could indicate a role for TGFβ signaling in the epicardium. Epicardial specific lineage trace models are necessary to demonstrate to what extend the defective formation of the ECM and cardiomyocyte proliferation caused by TGFβ inhibition are related to disrupted epicardial EMT and invasion in the wound area of the injured zebrafish heart. Important to note is that most of the discussed literature involves the zebrafish heart; a research model that, in contrast to the murine and human heart, has regenerative capacity. Although these results are highly valuable because they give an indication how regeneration should look like, they are not directly representative for processes in the mammalian heart.

### 7.2. Epicardial BMP Signaling in the Injured Heart

A role for BMP signaling has also been implied in cardiovascular disease extensively [137,138,139,140], although knowledge about BMP signaling specifically after myocardial infarction remains limited (reviewed in [141]). Within 1 day after cardiac injury in mouse hearts, *bmp2* ligand expression starts to increase and reaches a maximal level after 3 days where after expression declines. After 7 days, expression of other BMP ligands, such as *bmp4*, *bmp6* and *bmp10* appears. For the epicardium specifically, the presence of relevant signaling proteins was revealed in a spatially restricted RNA sequencing experiment [142]. Validation experiments showed that expression of BMP ligands *bmp2b* and *bmp7* were enhanced in the wound border zone and also in the epicardium covering the wound at 7 days post injury (dpi). In addition, single cell sequencing of the zebrafish epicardium identified *bmp4* as a specific epicardial marker of the adult zebrafish heart [143], suggesting that BMP4 has a role particularly in the epicardium. This epicardial *bmp4* expression in the zebrafish heart was enhanced after injury [143]. Moreover, epicardial expression of *bmpr1aa*, ortholog of BMP type I receptor ALK3, was enhanced in the viable myocardium and the epicardium 7 dpi. These expression levels indicate that BMP signaling can occur in the injured heart including the epicardium, which was confirmed by the presence of pSmad1/5/8 in the epicardial layer at 3 dpi [142]. When BMP signaling was inhibited in the zebrafish, an attenuated regeneration was observed, associated with compromised cardiomyocyte dedifferentiation and proliferation. Although no effect of BMP signaling inhibition on *wt1b* expressing epicardial cells was observed [142], a role for BMP signaling in the epicardium cannot be ruled out because in this study only re-expression of *wt1b* was assessed. Therefore, this only shows that BMP signaling does not interfere with epicardial activation, but effects on epicardial EMT and invasion were not described. Expression profiles of BMP receptors and downstream target proteins show that BMP signaling is activated in the epicardium after injury in the zebrafish heart (see Figure 3). To what extent this BMP signaling affects epicardial behavior and, as a result, cardiac regeneration is open for further research. In addition, mouse studies should reveal if epicardial BMP signaling described in the zebrafish is also present in the mammalian epicardium.

## 8. Epicardial TGFβ and BMP Signaling as a Target for Regenerative Therapy?

Given the recapitulation of the fetal processes after injury, knowledge obtained in developmental research can be applied to identify pathways that could be used for regeneration. This is particularly interesting in the context of the heart, because unlike most of the other organs in the human body, the heart has limited regenerative capacity. Due to the lack of sufficient cardiomyocyte proliferation, injured muscle tissue is replaced by scar tissue. This hampers the pump function of the heart and can ultimately lead to progressive heart failure. It is therefore essential to find novel therapeutic approaches to restore cardiac function in patients who suffered from a myocardial infarction. The epicardium could be a potential candidate for this, for its important contribution to the developing heart. It has already been shown that the epicardium becomes reactivated in the injured heart, but this response is sub-optimal compared to the contribution of the embryonic epicardium to the formation of the ventricle wall. For example, in the adult heart, epicardial cells display less migration into the injured myocardium [18], and fetal epicardial cells are more prone to undergo EMT [81]. Therefore, optimizing the epicardial response to repair could enhance cardiac function of an injured heart. This was indeed demonstrated in a mouse study where pre-treatment of the epicardium with thymosin β4 resulted in an increased epicardial activation, epicardial EMT and improved cardiac function after injury [57]. Hence, the main question that remains is how we can use the knowledge about TGFβ signaling in the epicardium of the developing and diseased heart to improve the repair of the injured adult heart. 

A growing body of evidence of in vitro studies indicates that increasing TGFβ and BMP signaling in the epicardium can increase the number of cells undergoing EMT. In the developing heart, promoting ALK5 signaling appears to be the most effective way to activate epicardial EMT and invasion. However, the pleiotropic and context-dependent nature of TGFβ signaling desires a more specific approach to prevent promotion of cardiac fibrosis, apoptosis and hypertrophy (reviewed in [132]). Therefore, it is preferable to look for a more specific target to kindly push ALK5 signaling in the right direction: the induction of epicardial EMT and invasion. In this regard, TGFβR3 is a potential target since it can modulate ALK5 signaling and is essential for invasion of fetal epicardial cells. To be able to pursue this line of research, it is important to study the role of TGFβR3 in the injured adult heart. It would be interesting to compare TGFβR3 activity between the fetal and adult epicardium to provide potential targets for regenerative therapy. Furthermore, for a more complete and robust view on what TGFβR3 does in the epicardium, it might be relevant to develop additional models, e.g., inducible TGFBR3−/− mouse models, to study its behavior since a lot of the current literature is depending on cell culture systems. A second way to target ALK5 signaling to repair the heart could be via TGFβ-activating proteins that are not a direct member of the TGFβ family but can regulate its activity. Of interest in the context of TGFβ and cardiac regeneration is thymosin β4, a peptide that has been shown to enhance TGFβ signaling [144]. Although debated [145], one study showed that a myocardium specific KO of thymosin β4 resulted in a cardiac phenotype that displays similarities to the *Alk5* KO embryonic heart, e.g., detached epicardium, non-compacted myocardium and defective smooth muscle cell coverage of coronary vessels [146]. Moreover, in the adult heart, it has been shown that priming the epicardium with thymosin β4 increases cardiac function after injury in a mouse model. This was associated with increased epicardial EMT [57]. Although indirectly, this suggests that enhancing TGFβ signaling has the potential to improve the contribution of the epicardium to repair of the heart. A third way to increase epicardial EMT and invasion could be to target downstream signaling of ALK5, e.g., VCAM-1 or Rho signaling. Both changes in cell-cell adhesion molecules and rearrangement of the cytoskeleton are essential for EMT and invasion of the embryonic epicardium (see Section 4.3). We anticipate that reducing epithelial cell-cell adhesion via VCAM-1 or E-cadherin, or enhancing cytoskeletal changes via Rho signaling could lower the threshold for a cell to undergo EMT. This would increase the number of epicardial cells that can invade into the heart, without the need for activation of the pleiotropic TGFβ pathway.

Another opportunity to activate the epicardium of the injured heart could be via BMP4 signaling. During development, BMP4 was shown to be vital for epicardial maturation [42]. Thus far, the exact nature and function of epicardial maturation is not extensively described, but the fact that two of the main epicardial specific proteins, WT1 [42] and TCF21 [147], are proposed to be involved in this process indicates that it is an essential part of epicardial development. Interestingly, in vivo studies in zebrafish have indicated that BMP4 is specifically upregulated in the epicardium of the injured heart, similar to cardiac development. Although not studied, this could indicate that epicardial maturation is also involved in the epicardium of the injured heart. For instance, BMP4 could be involved in the de-differentiation of the epicardium, enabling it to reactivate its fetal behavior. This is of particular interest, since this study was performed in zebrafish, a research model that, in contrast to the murine and human heart, has regenerative capacity. Although these results are not directly representative for processes in the human heart, they are highly valuable as they give an indication how regeneration could occur. Therefore, BMP4 and epicardial maturation could be an interesting research line for follow up.

To be able to investigate these mechanisms in epicardial EMT, our research group has developed an in vitro cell culture system that allows for a direct comparison of fetal and adult human primary epicardial cells. Interestingly, while adult cells need a trigger to undergo EMT, fetal epicardial cells are more active and need incubation with an ALK4/5/7 kinase inhibitor to prevent spontaneous EMT [81]. This observation is in line with the in vivo situation and therefore provides a relevant model to investigate how adult epicardial EMT and invasion can be improved, using fetal epicardial cells as a blue print.

## 9. Conclusions

In this review we aimed to provide a structured overview of the role of multiple ligands and receptors of the TGFβ family in epicardial behavior. In our opinion, the strength of this review is the structured and detailed overview on the potential power of the TGFβ pathway. This also presents a limitation of this manuscript; there are several other signaling pathways (including Wnt [58], FGF [48] and PDGF [82]) involved in epicardial behavior which are now underrepresented, but these have been reviewed extensively elsewhere [148,149]. Regarding the TGFβ family, including TGFβ and BMP signaling, we can conclude that these signaling cascades are essential for epicardial EMT and invasion in the developing heart. Specific epicardial KO of ALK5 and ALK3 in vivo show an impaired development of the heart and in vitro studies demonstrate that this is due to the inability of epicardial cells to undergo EMT and to invade underlying tissue. The most important receptor for epicardial EMT and invasion appears to be ALK5, the main TGFβ type I receptor. In the injured heart, more research is needed to draw conclusions regarding the role of the TGFβ family in the epicardial contribution to repair, but the presence of relevant ligands and receptors in the adult epicardium indicates that TGFβ and BMP signaling occurs. For regeneration, opportunities lie within finding specific approaches to activate TGFβ signaling in a controlled manner, which could be achieved via TGFβR3 or thymosin β4.
